# Effects of Elevated Temperature on the Susceptibility of Capsicum Plants to Capsicum Chlorosis Virus Infection

**DOI:** 10.3390/pathogens11020200

**Published:** 2022-02-02

**Authors:** Wei-An Tsai, Jonathan R. Shafiei-Peters, Neena Mitter, Ralf G. Dietzgen

**Affiliations:** Centre for Horticultural Science, Queensland Alliance for Agriculture and Food Innovation, The University of Queensland, St. Lucia, QLD 4072, Australia; w.tsai@uq.edu.au (W.-A.T.); jonathan.peters@uq.edu.au (J.R.S.-P.); n.mitter@uq.edu.au (N.M.)

**Keywords:** capsicum chlorosis virus, *Capsicum annuum*, elevated temperature, antiviral RNA silencing, recovery phenotype

## Abstract

Capsicum, an important vegetable crop in Queensland, Australia, is vulnerable to both elevated temperatures and capsicum chlorosis virus (CaCV). Thus, it is imperative to understand the genetic responses of capsicum plants (*Capsicum annuum*) to CaCV under elevated temperature conditions. Here, we challenged susceptible plants (cv. Yolo Wonder) with CaCV and investigated the effects of elevated temperature on symptom expression, the accumulation of virus-derived short interfering RNA (vsiRNA) and viral RNA, and the expression of plant defense-associated genes. CaCV-inoculated plants initially showed more severe symptoms and higher viral concentrations at a higher temperature (HT, 35 °C) than at ambient temperature (AT, 25 °C). However, symptom recovery and reduced viral RNA accumulation were seen in the CaCV-infected plants grown at HT at later stages of infection. We also observed that HT enhanced the accumulation of vsiRNAs and that, concurrently, RNA interference (RNAi)-related genes, including *Dicer-like2* (*DCL2*), *DCL4*, *RNA-dependent RNA polymerase 1* (*RdRp1*), *RdRp6*, and *Argonaute2* (*AGO2*), were upregulated early during infection. Moreover, continuous high levels of vsiRNAs were observed during later stages of CaCV infection at HT. Overall, our investigation suggests that HT facilitates CaCV replication during early infection stages. However, this appears to lead to an early onset of antiviral RNA silencing, resulting in a subsequent recovery from CaCV in systemic leaves.

## 1. Introduction

Global food production is projected to become increasingly insecure due to future climate change scenarios [1]. Among all environmental variables, warmer temperatures are one of the vital climate stressors that influence food security globally [1]. Rising temperatures can cause crop production losses by directly interfering with plant physiological processes, plant development, and reproduction [2,3,4]. This has been seen in several crops, such as bean (*Phaseolus vulgaris* L.) [5], cowpea (*Vigna unguiculata* (L.) Walp.) [6], corn (*Zea mays* L.) [7], tomato (*Solanum lycopersicum* L. Mill.) [8], and capsicum (*Capsicum annuum* L.) [9,10,11], which are sensitive to high temperature during their reproductive stages. Additionally, an increase in temperature also influences crop yield indirectly through changes in plant disease progression or insect pest biology [12,13,14,15,16].

The spread of plant virus diseases is one of the risks to crop production that can be affected by a changing climate. Virus diseases decrease the marketable quality of cultivated crops and result in yield losses [17]. It is particularly important that elevated temperatures are likely to make virus disease outbreaks more difficult to predict [18,19,20]. Capsicum chlorosis virus (CaCV), which causes a serious disease in major capsicum production areas in Australia, is a vector-borne virus that is mainly transmitted by three species of thrips, *Thrips palmi*, *Ceratothripoides sclaratris*, and *Frankliniella schultzei* [21,22]. The symptoms caused by CaCV on capsicum plants include chlorotic concentric ringspots, mottling spots, and deformation on leaves, as well as plant stunting [21,23]. Taxonomically, CaCV has been classified in the species *Capsicum chlorosis orthotospovirus*, genus *Orthotospovirus*, family *Tospoviridae*. CaCV is phylogenetically placed in the watermelon silver mottle virus (WSMoV) clade and is closely related to serogroup IV tospoviruses, including WSMoV and groundnut bud necrosis virus (GBNV) [24,25,26]. Similar to other tospoviruses, the CaCV genome consists of large (L), medium (M), and small (S) genomic RNA segments [26]. In the L segment, an RNA-dependent RNA polymerase (RdRp) is encoded in the negative sense. In the M and S segments, nonstructural viral movement protein (NSm) and viral silencing suppressor (VSR) protein (NSs), respectively, are encoded in viral sense polarity, and the structural Gn and Gc glycoproteins and the nucleocapsid (N) protein are encoded in the negative sense [26]. 

Several studies have shown that exposure to elevated temperatures may either enhance or reduce plant susceptibility to virus diseases [27,28,29,30,31,32,33,34,35,36,37]. Reduced plant susceptibility to viruses, caused by elevated temperatures, is associated with a phenomenon named “temperature masking” or “temperature-dependent symptom recovery” [38,39,40]. Symptom recovery is manifested as the emergence of young asymptomatic leaves following an initial systemic symptomatic infection [38]. This recovery phenomenon, due to elevated temperatures, was documented more than 60 years ago in several viral pathosystems, including cauliflower mosaic virus in cabbage, red raspberry mosaic in raspberries, and a virus complex in different plum varieties [41]. However, it was not until 2003 that a molecular link with RNA silencing was revealed [28]. RNA silencing, also called RNA interference (RNAi), is considered to be a major antiviral plant defense response mechanism [42,43,44]. The process of RNAi is triggered by double-stranded RNAs (dsRNAs) that are generated during virus replication, structured regions of RNA transcripts, or bidirectional transcription of overlapping reading frames [45,46]. After recognition of dsRNA structures, dicer-like 4 (DCL4)-, DCL2- or DCL3-mediated 21, 22, and 24 nt virus-derived short interfering RNAs (vsiRNAs) are produced and loaded onto RNA-induced silencing complexes (RISC). Based on different Argonaute (AGO) proteins that are incorporated into RISC, post transcriptional or transcriptional gene silencing is activated to silence viruses [47,48]. The involvement of RNAi in temperature-dependent symptom recovery was first reported in cymbidum ringspot virus (CymRSV)-infected *Nicotiana benthamiana*. In this study, CymRSV-infected plants had abundant vsiRNAs and were symptomless at 27 °C, while those plants failed to accumulate vsiRNAs and presented symptoms at 15 °C [28]. By silencing critical components of the RNAi process, RdRp6, DCL2, AGO2, AGO1, and HEN1 were suggested to participate in the high-temperature-stimulated RNAi pathway in different pathosystems [27,29,30]. Conversely, enhanced plant susceptibility to viruses caused by elevated temperatures is mainly associated with the differential expression of stress-related proteins [35,37,49]. Salicylic acid (SA) is a vital phytohormone that mediates multiple defense responses by regulating the expression of *pathogenesis-related* (*PR*) genes or enhancing RNAi [50,51,52,53]. SA-induced *PR-1b* and *PR-2* are highly expressed in potato virus Y (PVY)-infected thermo-tolerant/PVY-resistant potatoes at elevated temperatures. However, the expression of those *PR* genes is decreased in PVY-infected thermo-sensitive/PVY-susceptible potatoes grown at elevated temperature, resulting in increased virus accumulation and more severe symptoms [35]. 

In plant-tospovirus pathosystems, a change in susceptibility to virus infection is exhibited in different plant species grown at elevated temperatures. Tomato spotted wilt virus (TSWV) infection in tomato shows a higher replication at 20 °C, while symptoms are more severe at 36 °C [54,55]. GBNV infection in cowpea only shows a severe necrosis at higher temperatures (30 °C and 25 °C) but not at 20 °C and 15 °C [56]. On the contrary, the systemic infection of impatiens necrotic spot virus (INSV) in capsicum plants (*C. annuum*, *C. chinense* PI152225, and *C. chinense* PI159236) was completely blocked when they were kept at a constant elevated temperature of 33 °C [57]. 

Collectively, there is no one-size-fits-all viral pathosystem that can be used as a general model to illustrate the effects of elevated temperatures on plant–virus interactions. Since CaCV is a tospovirus that affects capsicum plants in geographically and environmentally diverse production areas, it is important to understand the effects of elevated temperatures on the susceptibility of capsicum to CaCV. In this study, we explore the genetic responses and susceptibility of capsicum plants to CaCV infection at an elevated temperature of 35 °C by analyzing the symptoms and expression of defense genes related to RNAi and resistance genes. Furthermore, the accumulation of viral RNAs and vsiRNAs are investigated to evaluate the involvement of antiviral RNA silencing in capsicum susceptibility to CaCV at high and ambient growing temperatures.

## 2. Results

### 2.1. Effect of Elevated Temperature on CaCV Infection in Susceptible Capsicum Plants

**Figure 1:**
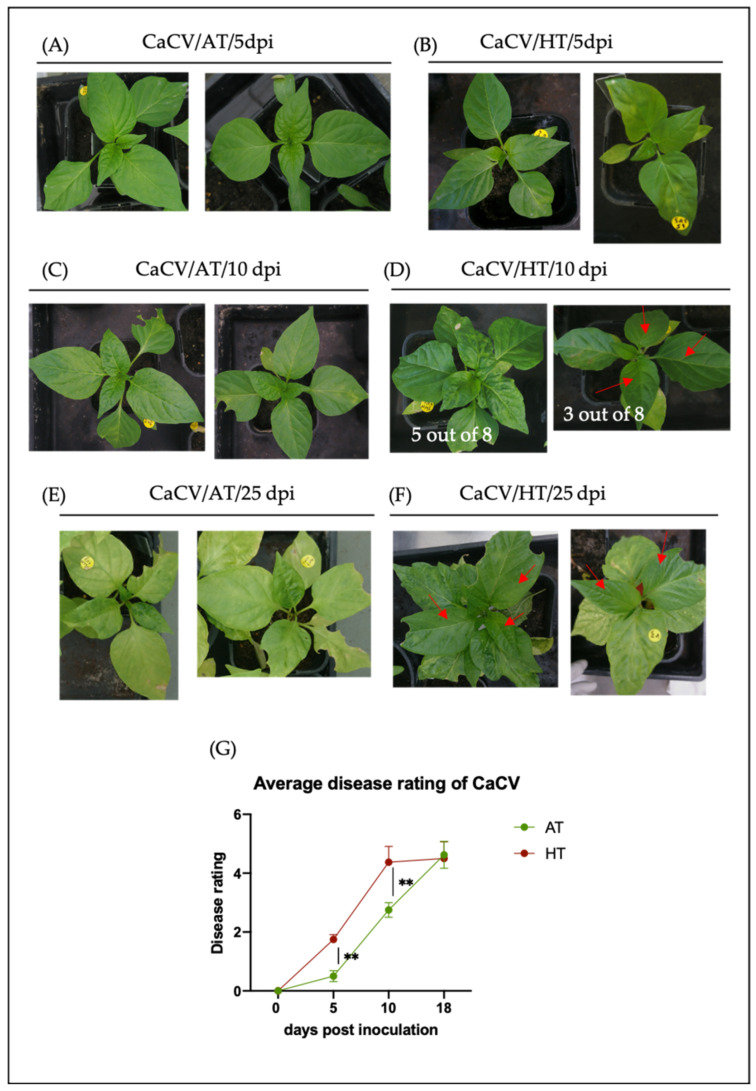
Effects of temperature on capsicum chlorosis virus (CaCV) infection in *Capsicum annuum* cv. Yolo Wonder. Representative symptoms of CaCV in infected capsicum plants grown at the ambient temperature (AT) of 25 °C at (**A**) 5 days post inoculation (dpi), (**C**) 10 dpi, and (**E**) 25 dpi in contrast to those plants grown at the higher temperature (HT) of 35 °C at (**B**) 5 dpi, (**D**) 10 dpi, and (**F**) 25 dpi. Recovered leaves that presented in CaCV-infected capsicum plants grown at HT at (**D**) 10 dpi (right panel) and (**F**) 25 dpi are indicated by red arrows. (**G**) Graph of the average disease ratings (Table 1) observed in CaCV-inoculated capsicum plants grown at AT or HT. The averages and standard errors are shown (*n* = 8). The significance of differences between CaCV-infected plants grown at AT and those grown at HT were assessed with Student’s *t*-test (** *p* < 0.01).

To investigate the effect of elevated temperature on symptom expression of CaCV-infected capsicum, susceptible plants were challenged with the combined stresses of virus infection and elevated temperature. Four-week-old plants were inoculated with CaCV, then half of the plants were transferred to high temperature (HT), and half remained at ambient temperature (AT). By 10 days post inoculation (dpi), all CaCV-infected plants grown at HT (Figure 1B,D) had more severe symptoms than those plants grown at AT (Figure 1A,C). Interestingly, a recovery phenotype manifested as mild systemic symptoms in newly emerged leaves in 3 out of 8 CaCV-infected plants grown at HT at 10 dpi (right panel of Figure 1D), while this recovery phenotype was seen later in the other 5 CaCV-infected plants grown at HT by 15, 18, or 21 dpi (Figure 1F). This slowed the increase in disease ratings of the CaCV-infected plants grown at HT after 10 dpi (Figure 1G). By 25 dpi, more expanded and less wrinkled newly developed leaves were observed in all CaCV-infected plants grown at HT (Figure 1F) than in those grown at AT (Figure 1E). In contrast to plants that were subjected to both CaCV infection and HT, the plants that were challenged with CaCV at AT developed mild symptoms in the early stages of infection but showed more severe symptoms by 25 dpi (Figure 1A,C,E,G).

### 2.2. Effect of Elevated Temperature on CaCV RNA and vsiRNA Accumulation in Capsicum Plants

**Figure 2:**
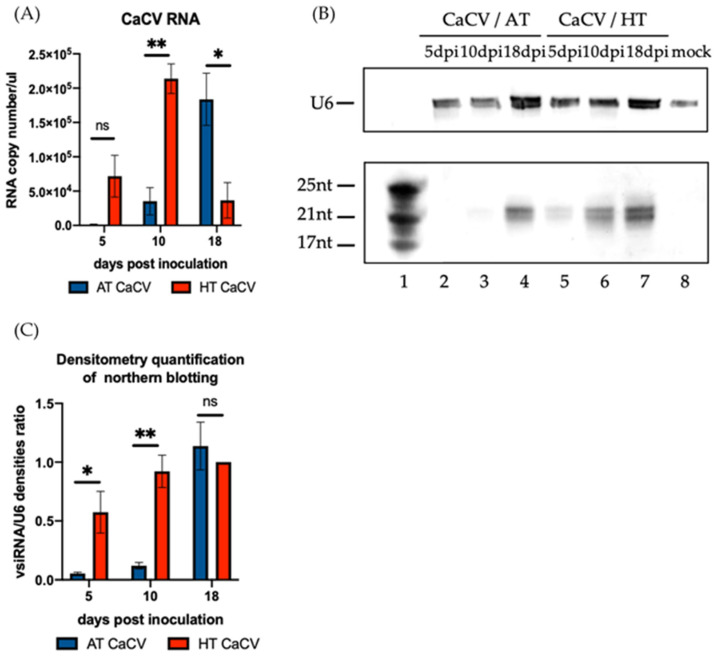
Effect of elevated temperature on the accumulation of capsicum chlorosis virus (CaCV) RNA and virus-derived short interfering RNA (vsiRNA) in CaCV-infected capsicum plants grown at ambient temperature (AT) or high temperature (HT) at 5, 10, and 18 days post inoculation (dpi). RNA accumulation was measured in inoculated leaves at 5 dpi and in systemic leaves at 10 and 18 dpi. (**A**) The absolute quantification of CaCV RNA measured by real time RT-PCR in samples of CaCV-infected capsicum plants grown at AT or HT. The bars represent the mean (±the standard error of the mean) of six biological replicates. (**B**) The detection of CaCV vsiRNAs by northern blot hybridizations in CaCV-infected capsicum plants grown at AT (lane 2 to lane 4) or HT (lane 5 to lane 7). U6 was used as an internal control and a mock-inoculated sample was used as a negative control. Small RNA size markers are shown in lane 1. (**C**) The relative accumulation levels of CaCV vsiRNA in CaCV-infected capsicum plants grown at AT or HT were quantified by measuring the signal strength using Image J. The band intensity of the vsiRNAs was calibrated with that of the U6 internal control. The bars represent the mean (±the standard error of the mean) of four biological replicates. The significance of the differences between treatments was assessed with Student’s *t*-test (* *p* < 0.05; ** *p* < 0.01; ns, not significant).

Accumulation of CaCV RNAs was measured at 5, 10, and 18 dpi by absolute quantitation through real time RT-PCR analysis. Six biological replicates for each treatment were collected from eight plants used for phenotypic analysis. A standard curve with an r-square (R^2^) value of 0.99583 was generated for determining the abundance of viral RNA (Figure S1). At 5 dpi, the differences between the viral RNA levels in CaCV-infected plants grown at HT and AT were not statistically significant, with a *p*-value of 0.08 (Figure 2A). At 10 dpi, the viral RNA accumulation in systemic leaves was significantly higher in the CaCV-infected plants grown at HT than those grown at AT (Figure 2A). At 18 dpi, less viral RNA had accumulated in the plants grown at HT, while a significant increase in viral RNA accumulation had occurred in the plants grown at AT (Figure 2A). Considering that virus-induced RNA silencing plays an important role in temperature-dependent symptom recovery [27,28,29,30,31,32,33,34], the accumulation of vsiRNAs was examined at the same time points of 5, 10, and 18 dpi. Four biological replicates, which were selected from the six RNA samples used for viral RNA quantification, were examined for vsiRNA accumulation. Among those four treated with CaCV and HT, based on symptoms, two had recovered from CaCV at 18 dpi, while the remaining two had recovered from virus infection at 21 dpi. A northern blot analysis showed that the amount of CaCV-derived 21 and 22 nt siRNAs in the plants grown at HT gradually increased from 5 dpi to 10 dpi and remained highly abundant at 18 dpi (Figure 2B,C). Differences of vsiRNAs accumulation were observed in plants grown at the two different temperatures at 5 and 10 dpi. At these time points, vsi-RNAs in plants grown at HT were more abundant than those grown at AT. However, similar high amounts of CaCV vsiRNAs were detected in plants grown at AT and HT at 18 dpi (Figure 2B,C). These results suggest that the levels of CaCV RNAs and vsiRNAs were likely linked to the recovery phenotype and the early symptom development triggered by elevated temperature.

### 2.3. Differential Expression of RNAi-Associated Genes in the Capsicum Response to CaCV Infection at High and Ambient Temperature

**Figure 3:**
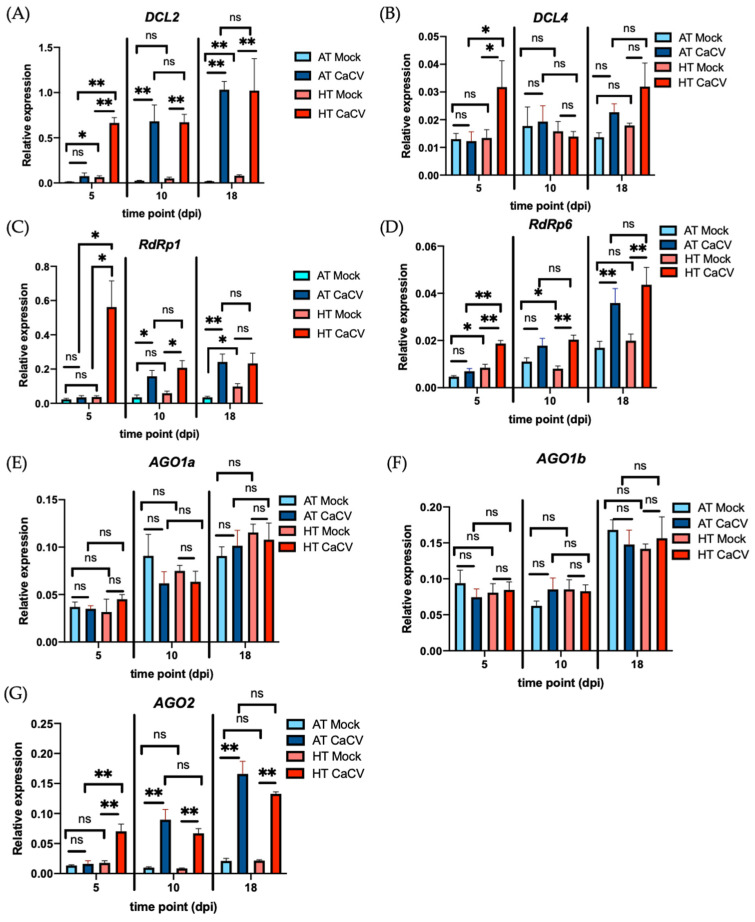
Effect of high (HT) and ambient (AT) temperatures on the expression of selected RNAi-associated genes in capsicum chlorosis virus (CaCV)-infected and mock-inoculated capsicum plants at 5 days post inoculation (dpi), 10, and 18 dpi. The relative gene expression was measured by RT-qPCR in inoculated leaves at 5 dpi and systemic leaves at 10 and 18 dpi. The expression of (**A**) *DCL2*, (**B**) *DCL4*, (**C**) *RdRp1*, (**D**) *RdRp6*, (**E**) *AGO1a*, (**F**) *AGO1b*, and (**G**) *AGO2* was analyzed with the 2^−ΔCt^ method. *Actin* was used as internal control. The bars represent the means (±standard the error of the mean) of six biological replicates. The significance of the differences between treatments was assessed with Student’s *t*-test (* *p* < 0.05; ** *p* < 0.01; ns, not significant).

To elucidate the molecular mechanisms involved in plant recovery from CaCV infection at HT, the expression of selected RNAi-associated genes, including *DCL2*, *RdRp6*, *AGO1a*, *AGO1b*, and *AGO2*, was analyzed, based on their known association with temperature-dependent recovery [27,29,30]. Additionally, *DCL4* was selected due to its major involvement in vsiRNA generation, and *RdRp1* was selected due to its known involvement in capsicum resistance to virus disease [58]. The relative expression levels of the RNAi-associated genes at 5 dpi in inoculated leaves showed that the *DCL2*, *DCL4*, *RdRp1*, *RdRp6*, and *AGO2* transcripts were significantly increased at HT in CaCV-infected plants compared to mock-inoculated plants (Figure 3A–D,G). However, the levels of those transcripts in CaCV-infected plants grown at AT were low and were comparable to those in mock-inoculated plants (Figure 3A–D,G). This data indicates that at 5 dpi the transcript levels of *DCL2*, *DCL4*, *RdRp1*, *RdRp6*, and *AGO2* in the virus-inoculated leaves were significantly higher in CaCV-infected plants grown at HT versus those grown at AT (Figure 3A–D,G). Unlike the other RNAi-associated genes analyzed, the capsicum transcript levels of *AGO1a* and *AGO1b* showed no significant differences in relation to CaCV infection or plant growth temperature at 5 dpi (Figure 3E,F). 

At 10 dpi in systemic leaves, the transcript levels of *DCL2*, *RdRp1*, and *AGO2* were upregulated in CaCV-infected plants compared to mock-inoculated plants grown at both HT and AT (Figure 3A,C,G). Conversely, the transcript levels of *RdRp6* were upregulated by CaCV infection at only HT but not AT (Figure 3D), while the transcript levels of *DCL4*, *AGO1a*, and *AGO1b* remained unchanged across all treatments (Figure 3B,E,F). Although increased levels of the *DCL2*, *RdRp1*, *RdRp6*, and *AGO2* transcripts were found at 10 dpi in CaCV-infected plants compared to mock-inoculated plants grown at HT (Figure 3A,C,D,G), there were no statistically significant differences in the expression of these genes between CaCV-infected plants grown at HT or at AT. 

At 18 dpi, the levels of all selected RNAi-associated transcripts in the CaCV-infected plants grown at HT were comparable to those in plants grown at AT (Figure 3). In comparison to the mock-inoculated plants, transcript levels of *DCL2*, *RdRp6*, and *AGO2* were elevated by CaCV infection at both AT and HT at 18 dpi (Figure 3A,D,G). Unlike those three genes, the transcript level of *RdRp1* was induced by CaCV infection at only AT but not at HT (Figure 3C). This may be due to the increased level of *RdRp1* in mock-inoculated plants grown at HT compared to AT. Furthermore, the transcript levels of *DCL4*, *AGO1a*, and *AGO1b* remained unchanged in plants infected with CaCV grown at either AT or HT (Figure 3B,E,F). 

In the absence of CaCV infection, *DCL2*, *RdRp1*, and *RdRp6* were differentially affected by temperature (Figure 3A,C,D). Slight increases in the levels of the *DCL2* transcripts were observed at 5 dpi and 18 dpi in the mock-inoculated plants grown at HT compared to those grown at AT (Figure 3A). A slightly increased level of *RdRp1* was seen at 18 dpi in the mock-inoculated plants grown at HT compared to AT (Figure 3C). Furthermore, slightly increased or reduced levels of *RdRp6* were seen in the mock-inoculated plants grown at HT compared to AT, at 5 dpi and 10 dpi, respectively (Figure 3D). 

**Figure 4:**
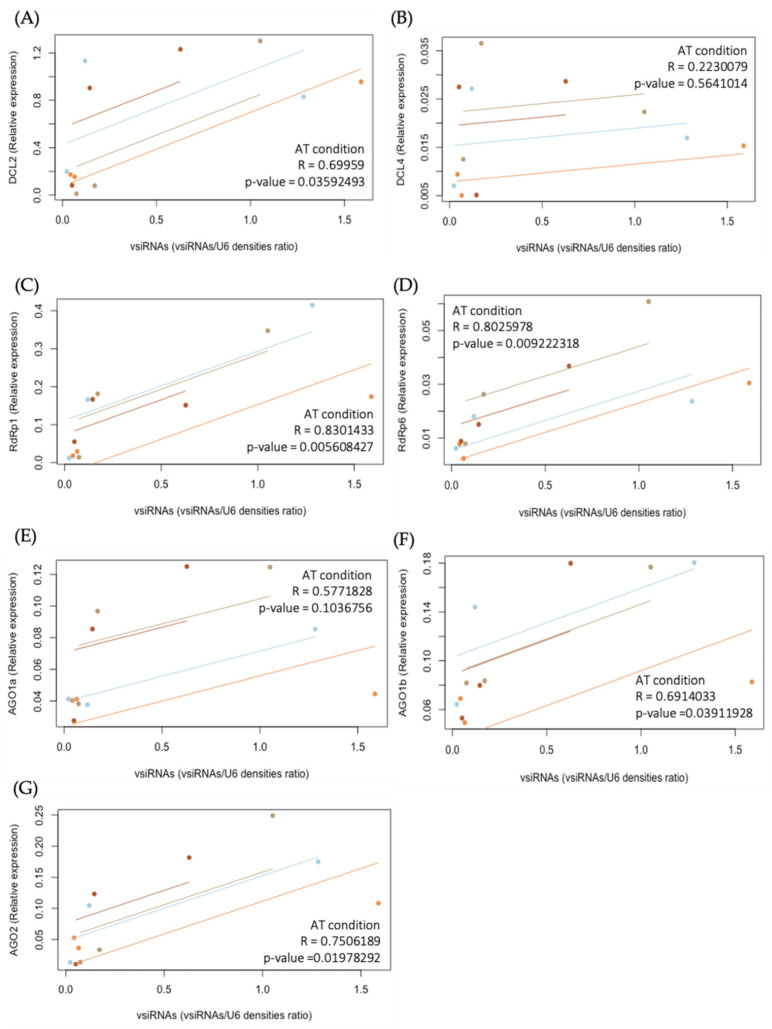
Investigation of the correlations between the repeatedly measured expression of RNAi-associated genes: (**A**) *DCL2*, (**B**) *DCL4*, (**C**) *RdRp1*, (**D**) *RdRp6*, (**E**) *AGO1a*, (**F**) *AGO1b*, and (**G**) *AGO2* and the abundance of virus-derived short interfering RNAs (vsiRNAs) at ambient temperature (AT) over time. Scatter plots for the repeated measures correlation (rmcorr) showed a positive correlation between the abundance of vsiRNAs and the expressions of (**A**) *DCL2*, (**C**) *RdRp1*, (**D**) *RdRp6*, (**F**) *AGO1b*, and (**G**) *AGO2* over time. No correlation was shown between the abundance of vsiRNAs and the expressions of (**B**) *DCL4*, and (**E**) *AGO1a*. The different colors represent different individuals of four biological replicates. The R values represent strong correlations when close to 1 and negative correlations when close to −1. A significant correlation is indicated if the *p* value is <0.05.

Taken together, these results suggest that the effect of CaCV infection on expression of *DCL2*, *DCL4*, *RdRp1*, *RdRp6*, and *AGO2* genes is more pronounced than the effect of plant growth temperature. Moreover, high temperature induces higher transcript accumulation levels of *DCL2*, *DCL4*, *RdRp1*, *RdRp6*, and *AGO2* during the early stages of CaCV infection (Figure 3A–D,G), which showed a similar pattern to the higher accumulation of vsiRNAs at HT at 5 dpi (Figure 2D). To investigate if there is a correlation between gene expression and vsiRNA accumulation, the within-individual correlation for multiple individuals at three time points was assessed by rmcorr (Figure 4 and Figure S2). The expression of *DCL2*, *RdRp1*, *RdRp6*, *AGO1b*, and *AGO2* was significantly correlated with the accumulation of vsiRNAs at AT over time, with r values of 0.70, 0.83, 0.80, 0.69, and 0.75, respectively (Figure 4A,C,D,F,G). However, only the expression of *RdRp6* was correlated with the accumulation of vsiRNAs over time at both AT and HT (Figure S2).

### 2.4. RdRp1 Is Involved in N. Benthamiana tolerance to CaCV Infection at High and Ambient Temperatures

Symptom recovery has been considered as an inducible form of tolerance [38]. *N. benthamiana* laboratory isolate (LAB), which lacks a functional *RdRp1* gene [59], was susceptible to tospoviruses (Fletcher, S. J. et al. unpublished data) and many other viruses [59]. On the other hand, *N. benthamiana* Western Australia isolate (WA), which has a functional *RdRp1* gene [59], was resistant to tospoviruses (Fletcher, S. J. et al. unpublished data) and a range of other viruses [59]. Here, LAB and WA plants were used to examine the involvement of *RdRp1* in host plant tolerance to CaCV at both AT and HT. At 10dpi, the LAB and WA plants that were challenged with CaCV displayed comparable mild yellowing symptoms at AT (Figure S3A). At 20 dpi, the CaCV-infected LAB plants developed severe necrosis, while the CaCV-infected WA plants emerged newly recovered leaves at AT (Figure S3C). Interestingly, more severe symptoms were observed on all the CaCV-infected LAB plants grown at HT compared to those grown at AT at both 10 dpi and 20 dpi (Figure S3). By contrast, only slightly enhanced symptom development at HT was seen on the CaCV-infected WA plants at 20 dpi (Figure S3C,D). This suggests that functional RdRp1 is involved in plant tolerance to CaCV infection not only at AT but also at HT. 

### 2.5. Differential Expression of Resistance-Associated Genes in Capsicum Response to CaCV Infection at High and Ambient Temperatures

To analyze the involvement of pathogen resistance (R)-associated genes in the defense of capsicum plants against CaCV at HT, host genes were selected, based on a previous differential expression study that compared the CaCV-resistant *C. annuum* x *C. chinense* challenged with CaCV vs the CaCV-susceptible *C. annuum* challenged with CaCV [23]. *Suppressor of the G2 allele of skp1 (SGT1*) and *Capsicum resistance gene 2* (*CaRg2*) were selected, due to a significant transcript upregulation in the CaCV-resistant line compared to the susceptible line. This upregulation may indicate important contributions of these genes to plant defense against CaCV [23]. *Phytochrome interacting factor 4 (PIF4)*, a negative regulator of R-gene-mediated resistance [60], was selected because of significant transcript down-regulation in the CaCV-resistant line compared to the susceptible line. Moreover, PIF4 was downregulated in the CaCV-resistant capsicum when challenged by CaCV, but its expression remained unchanged in the CaCV-susceptible capsicum when challenged by CaCV, indicating that PIF4 may be involved in the susceptibility of capsicum plants to CaCV infection [23]. 

**Figure 5:**
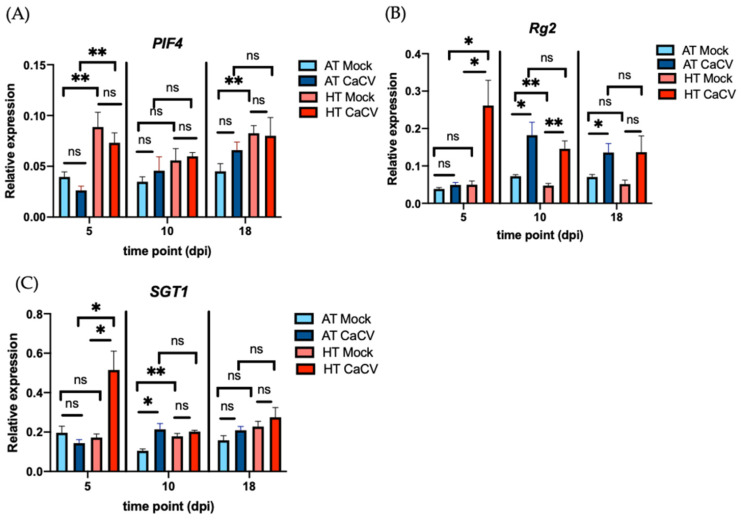
Effect of ambient (AT) and high (HT) temperature on the expression of the selected resistance-associated genes in capsicum chlorosis virus (CaCV)-infected and mock-inoculated capsicum plants at 5 days post inoculation (dpi), 10, and 18 dpi. Gene expression was measured by RT-qPCR in inoculated leaves at 5 dpi and systemic leaves at 10 and 18 dpi. The relative expression of the (**A**) *PIF4*, (**B**) *CaRg2*, and (**C**) *SGT1* genes was analyzed with the 2^−ΔCt^ method. *Actin* was used as an internal control. The bars represent the means (±standard the error of the mean) of six biological replicates. The significance of the differences between treatments was assessed with Student’s *t*-test (* *p* < 0.05; ** *p* < 0.01; ns, not significant).

In CaCV-inoculated capsicum leaves at 5 dpi, the levels of all three transcripts, *PIF4*, *CaRg2*, and *SGT1*, were significantly higher in plants grown at HT compared to AT (Figure 5). However, only *CaRg2* and *SGT1* were upregulated at 5 dpi in CaCV-infected plants compared to mock-inoculated plants at HT (Figure 5B,C). The expression of *PIF4* at 5 dpi in mock-inoculated plants was significantly enhanced at HT (Figure 5A), while the expression of the other two remained unchanged. At 10 dpi in systemic leaves, no significant differences of the expression of *PIF4* were observed among all treatments (Figure 5A). Unlike *PIF4*, the expression of *CaRg2* was enhanced by CaCV infection at both HT and AT (Figure 5B), while the expression of *SGT1* was enhanced by CaCV infection only at AT (Figure 5C). An increased level of the *SGT1* transcripts and a decreased level of the *CaRg2* transcripts were observed in the mock-inoculated plants grown at AT compared to those grown at HT (Figure 5B,C). At 18 dpi, the expression of *PIF4* was significantly enhanced at HT in the mock-inoculated plants, and the expression of *CaRg2* was significantly enhanced by virus infection at AT (Figure 5A,B). Taken together, these results suggest that the *PIF4* transcripts are upregulated by elevated temperature, whereas the *CaRg2* transcripts are induced by CaCV infection. However, the previously reported inhibition of plant defense gene expression by *PIF4* [61] was not observed for *CaRg2* in our study.

## 3. Discussion

Temperature increases have been reported to affect plant susceptibility to virus diseases in various studies. In some cases, higher temperatures enhance plant tolerance to virus diseases during the late stages of infection, leading to a recovery from symptoms in newly emerged leaves [27,28,29,30,31,32,33,34]. In other cases, higher temperatures weaken plant defense responses or increase virus accumulation, resulting in more severe symptoms at both the early and late stages of infection [35,37]. In the present study, capsicum plants that were infected with CaCV initially developed more intense symptoms at HT than at AT. However, during the later stages of infection, new leaves recovered from systemic symptoms in the CaCV-infected capsicum grown at HT but not at AT, which indicates a recovery response due to HT. 

The effects of elevated temperatures on tospovirus symptomatology have been shown to vary, depending on different combinations of tospoviruses and their host plant species [56,57,62]. For example, the spread and symptom severity of GBNV in cowpea plants increased at higher temperatures (30 and 25 °C) compared to 20 and 15 °C [56]. At higher temperatures, the viral titer and RNA of GBNV, as well as hydrogen peroxide levels, were increased in inoculated leaves (at 4 and 8 dpi) and systemic leaves (at 24 dpi), which resulted in severe necrosis [56]. Similarly, more severe symptoms were observed in TSWV-infected *Nicotiana tabacum*, *Physalis ixocarpa*, and *Datura stramonium* at higher temperature (29/24 °C, day/night) compared to 23/18 °C, day/night [54]. However, the earlier accumulation of TSWV in whole plants, which was favored by higher temperature, was only observed in tobacco. This suggests that for TSWV, symptom expression at different temperatures is not necessarily correlated with virus accumulation [54]. Unlike the reports on GBNV-cowpea and TSWV-tobacco pathosystems, where elevated temperatures made plants more susceptible to tospoviruses, the symptoms of TSWV on new leaves of *Ficus* spp. were attenuated at higher temperature (35 °C) but reappeared at ambient temperature (25 °C) [62]. Furthermore, a constant high temperature of 33 °C led to a complete block of the systemic movement and symptom development of INSV in capsicum plants (*C. annuum*, *C. chinense* PI152225, and *C. chinense* PI159236) [57]. In our work, the symptoms of CaCV on newly emerged capsicum leaves were attenuated at 35 °C, similar to the effect of high temperature on TSWV-ficus and INSV-capsicum pathosystems. However, rather than completely inhibiting CaCV spreading from inoculated leaves to systemic leaves, higher temperature caused a decrease in CaCV RNA accumulation in systemic leaves during the late stages of infection. This suggests that the mechanisms involved in temperature-dependent symptom attenuation seen in CaCV-infected capsicum may be different to those involved in INSV-infected capsicum. Since the potential mechanism involved in symptom attenuation at high temperature in TSWV-infected *Ficus* spp. has not been investigated further, this pathosystem cannot provide insights for our study. Nevertheless, the mechanisms involved in symptom recovery in many other plant viral pathosystems [27,28,29,30,31,32,33,34] may provide some insights into the recovery phenotype seen in CaCV-infected capsicum at elevated temperature.

Antiviral RNAi appears to be the best-documented mechanism involved in temperature-dependent symptom recovery to date [27,28,29,30,31,32,33,34]. The accumulation of vsiRNAs has been validated to be essential for RNAi-mediated symptom recovery at elevated temperatures in many cases [28,29,30,32,33,34]. This agrees with our finding that vsiRNA levels were always higher at HT than at AT in the CaCV-inoculated and systemic leaves at 5 and 10 dpi. Even though the accumulation of vsiRNAs is essential for recovery, a concomitant viral nucleic acid reduction is not strictly required [38]. A lower viral RNA accumulation is associated with RNAi-mediated recovery caused by elevated temperatures in some cases [27,31,32,33]. For example, milder symptoms and reduced viral RNA levels were shown in turnip crinkle virus (TCV)-infected *N. benthamiana* plants at 27 °C compared to 21 °C [27]. However, unlike the TCV-infected *N. benthamiana* that displayed consistently fewer severe symptoms at HT, TCV-infected *Arabidopsis thaliana* showed more severe symptoms and a higher level of viral RNA during the early stages of infection [29]. Since the level of viral RNA was examined prior to the initiation of plant recovery, the higher viral RNA accumulation in TCV-infected *A. thaliana* may have resulted from more pronounced TCV RNA replication, due to elevated temperature during the early stages of infection. A faster accumulation of viral RNA during the early stages of infection, caused by elevated temperatures, was also reported in tomato ringspot virus (ToRSV)-infected *N. benthamiana* [30]. Notably, a similar level of RNA2 but a lower level of RNA2-encoded proteins was observed at 27 °C when compared to those at 21 °C after the initiation of a temperature-dependent symptom recovery in ToRSV-infected *N. benthamiana* [30]. In our study, more severe symptoms, accompanied by a higher level of viral RNA, were observed during the early stages of CaCV infection at HT compared to AT. This is similar to the TCV-infected *A. thaliana* and ToRSV-infected *N. benthamiana*, which showed a faster accumulation of viral RNA prior to the initiation of symptom recovery at HT. Interestingly, higher temperature caused a decline in the viral RNA levels in recovered leaves during the late stages of CaCV infection, which differs from the result of ToRSV-infected *N. benthamiana* but is similar to that of TCV-infected *N. benthamiana* at HT. Overall, the finding of a decrease in viral RNA levels in recovered leaves, together with that of continuously high vsiRNA levels, implies that RNAi is likely involved in the recovery phenotype seen in CaCV-infected capsicum at HT. 

Recently, several critical components involved in RNAi pathways, such as RdRp6, DCL2, AGO2, HEN1, and AGO1, have been reported to play important roles in temperature-dependent symptom recovery in a number of pathosystems. The silencing of RdRp6 prevents temperature-dependent symptom recovery in TCV-infected *N. benthamiana* [27]. Knocking down *DCL2*, *AGO2*, or *HEN1* gene expression thwarts the survival of TCV-infected *A. thaliana* at 26 °C [29]. In contrast, the silencing of *AGO1* instead of *AGO2* in ToRSV-infected *N. benthamiana* was shown to prevent symptom recovery at 27 °C [63]. To better understand the molecular link between elevated temperature and capsicum susceptibility to CaCV infection, we investigated the expression of those RNAi-related genes during the dual stresses of CaCV infection and HT. The transcript sequences of those genes in capsicum were based on our previous transcriptome analysis of CaCV-infected capsicum [23] and a study of *RdRp*, *DCL*, and *AGO* genes in capsicum [64]. The *HEN1* gene was excluded, due to a lack of its analysis in capsicum. The *RdRp1* gene was included, due to the evidence of its involvement in capsicum resistance to TMV [58]. The *DCL4* gene was included, since both 21 and 22 nt vsiRNAs accumulated equally in the CaCV-infected plants grown at HT or at AT. Except for *AGO1a* and *AGO1b*, we found that at 5 dpi, the expression of *RdRp1*, *RdRp6*, *DCL2*, *DCL4*, and *AGO2*, was induced by CaCV infection only at HT but not at AT, and this was associated with elevated levels of vsiRNA. In agreement with previous studies [29,30,32], this suggests that elevated temperature may trigger an early onset of RNA silencing, leading to increased vsiRNA accumulation. At 10 dpi in systemic leaves, a significantly higher accumulation of vsiRNA and viral RNAs were observed at HT; however, the expression of all selected RNAi-associated genes was similar in CaCV-infected plants grown at AT and those grown at HT. We speculate that this may be due to CaCV accumulation reaching a threshold in infected plants grown at either temperature, which may trigger a similar upregulation of RNAi-associated genes. Another notable finding is the apparent association between the expression pattern of *RdRp6* and *AGO2*, the levels of vsiRNAs, and the levels of viral RNAs in the plants infected with CaCV at 10 and 18 dpi. At 18 dpi, in recovered leaves, the expression of RNAi-associated genes was similar in CaCV-infected plants grown at AT and at HT. However, when comparing the transcript levels of RNAi-associated genes at 10 dpi with 18 dpi, a significant temperature-independent increase of the *RdRp6* and *AGO2* transcripts was detected in the CaCV-infected plants. Moreover, the expression of *RdRp6* was found to be positively correlated with the abundance of vsiRNAs over time at both HT and AT. RdRp6 is an important component for secondary vsiRNA production during RNA virus infections [65,66,67,68]. RdRps are also important for the spread of silencing signals [69]. Therefore, an increase in the transcript levels of *RdRp6* and its positive correlation with vsiRNA abundance may explain the marked increase in vsiRNA accumulation from 10 to 18 dpi in the CaCV-infected plants grown at AT. Furthermore, the increased *RdRp6* transcript levels may contribute to the continuously high levels of vsiRNAs in the CaCV-infected plants grown at HT from 10 to 18 dpi. Interestingly, rather than plateauing from 10 to 18 dpi like the levels of vsiRNAs, a marked reduction in the viral RNA levels was observed in the CaCV-infected plants grown at HT. Other than RdRp6, which may contribute to symptom recovery at HT, RdRp1 may also be critical for vsiRNA accumulation and symptom recovery, according to the data obtained by comparing symptom expressions of *N. benthamiana* WA plants, which harbor a functional RdRp1, and LAB, which lack a functional RdRp1. At 20 dpi, the CaCV-infected LAB plants displayed more severe symptoms at both HT and AT, whereas the CaCV-infected WA plants were tolerant to the infection at either temperature. Furthermore, a positive correlation between vsiRNA abundance and the expression of *RdRp1* was found in the CaCV-infected capsicum plants grown at AT. Taken together, our data suggest that elevated plant growing temperature accelerates CaCV RNA replication early during infection, thereby leading to earlier development of the symptoms of infection. However, the higher viral RNA load may trigger an early onset of RNA silencing and an early accumulation of vsiRNA, which, consequently, attenuate virus replication.

Since weakened plant defense responses have also been reported to be associated with enhanced plant susceptibility to viruses at elevated temperatures [35,36,37,49], we investigated if R gene-associated genes may be involved in the differential symptom expression in CaCV-infected plants induced by elevated temperature. We selected three genes, based on our previous differential expression analysis of CaCV-resistant and -susceptible capsicum challenged with CaCV. *SGT1* and *CaRg2* were selected, based on preliminary evidence of their involvement in plant defense against CaCV [23]. *PIF4* was selected, due to its probable positive association with plant susceptibility to CaCV [23]. At 5 dpi, the transcript levels of both *CaRg2* and *SGT1* were higher in the CaCV-infected plants grown at HT than at AT. However, the elevated expression of these genes at HT was unexpected, since the symptoms were more severe at HT than at AT. At 10 and 18 dpi, no significant difference in the expression of *CaRg2* and *SGT1* was observed between the CaCV-infected plants grown at HT and AT, while *CaRg2* was upregulated at both temperatures. While this result suggests that CaCV accumulation may trigger the accumulation of *CaRg2* transcripts, the association of *CaRg2* and *SGT1* with capsicum susceptibility to CaCV remains inconclusive. 

PIF4 is a negative regulator of plant resistance responses. At 27 or 28 °C, light receptor phytochrome B switches to the inactive form, which contributes to the enhanced accumulation of PIF4 [70]. Moreover, PIF4 inhibits the expression of defense-related genes, which leads to increased susceptibility of *A. thaliana* to *P. syringae* at 27 °C [60,61]. In this study, we found the expression of *PIF4* was induced by HT, either in the absence or presence of CaCV, at 5 dpi. Notably, the transcript level of *PIF4* at 18 dpi was higher in mock-inoculated plants grown at HT than at AT, while it was similar in the CaCV-infected plants grown at either temperature. This suggests that the expression levels of *PIF4* reflect the more severe symptoms seen at 5 dpi and the attenuated symptoms seen at 18 dpi. However, further experiments are required to clarify the involvement of *CaRg2* and *SGT1* in temperature-dependent symptom recovery.

Overall, based on the observed disease phenotype, viral RNA and vsiRNA accumulation, and gene expression analysis, we conclude that RNAi is likely involved in the temperature-dependent recovery observed in CaCV-infected capsicum.

## 4. Materials and Methods

### 4.1. Plant and Virus Materials, Growth Conditions, and High Temperature Treatment

*C. annuum* cv. Yolo Wonder plants were grown in a glasshouse at an AT of approximately 25 °C. Seedlings aged 4–5 weeks with 3 true leaves were mechanically inoculated with CaCV isolate QLD 3432 [26]. The inoculum was prepared in a mortar/pestle by grinding fresh CaCV-infected symptomatic capsicum leaves in 10 mM phosphate buffer, pH 7.6, with freshly added 20 mM sodium sulfite. The mock treatment used buffer only. The inoculum and buffer were rubbed onto the carborundum-dusted first two leaves and two cotyledons. Mock- and CaCV-inoculated plants were grown at AT for 1 day prior to half of each plant group being transferred to a growth cabinet with a HT of 35 °C/30 °C (16 h day/8 h night), a relative humidity of 60% and a light intensity of 230 μmol·m^−2^·s^−1^. *N. benthamiana* LAB and WA plants were provided by the Peter Waterhouse laboratory at the Queensland University of Technology. Seedlings were grown in a growth cabinet at an AT of 25 °C/20 °C (16 h day/8 h night) and were inoculated with CaCV at 4–5 true leaf stage. The methods of the mechanical inoculation and temperature treatments were the same as mentioned above.

### 4.2. Symptom Severity Rating of CaCV-Infected Capsicum Plants

Symptoms were recorded for up to 25 dpi. At the early stages of CaCV infection, the severity of symptoms was categorized into 7 levels, and 0–6 symptom scores were assigned, as defined in Table 1. At later stages of CaCV infection, the dpi at which plants recovered from symptoms was recorded.

### 4.3. RNA Extraction and cDNA Synthesis

Leaf disks (1 cm diameter) of mock-inoculated and CaCV-inoculated plants grown at HT and AT were collected for RNA isolation. Three leaf disks were sampled from inoculated leaves at 5 dpi, and another three leaf disks were sampled from the third leaf from the top at 10 dpi and 18 dpi, respectively, and immediately placed in liquid nitrogen for subsequent storage at −80 °C. Total RNA was extracted by using TRIsure reagent (Bioline, London, UK) according to manufacturer’s instructions. The isolated total RNA was then treated with TURBO™ DNase to remove contaminating DNA using the TURBO DNA free kit (Invitrogen, Waltham, MA, USA). Complementary DNA was synthesized using a SensiFAST™ cDNA Synthesis Kit (Bioline, London, UK), following manufacturer’s instructions, with reaction conditions of 25 °C for 10 min, 42 °C for 15 min, and 85 °C for 5 min.

### 4.4. Plasmid and Standard Curve Construction

To generate a standard curve for the absolute quantification of viral RNA, a recombinant plasmid containing the viral RNA target region was constructed. First, cDNA was synthesized from an RNA extract from CaCV-infected capsicum plants for use as a PCR template. The CaCV intergenic region (IR) of the S segment was then amplified by PCR using Phusion High-Fidelity DNA Polymerase (New England BioLabs, Ipswich, MA, USA) with the primers CaCV_IRF and CaCV_IRR (Table 2). The primers were designed from the genome sequence of CaCV isolate QLD 3432 (Widana Gamage et al., 2015). All primers were designed using Geneious Prime (Version 2019.1.3). The resulting 795 bp amplicon was A-tailed, purified from 1% agarose gel by the Wizard^®^ SV gel and PCR clean-up system (Promega, Madison, WI, USA), and was cloned into the pGEM^®^-T Easy vector (Promega). The recombinant plasmids were linearized by *Nde*I (New England BioLabs, Ipswich, MA, USA) and purified using Wizard SV gel and PCR clean-up system. The DNA concentration was measured by a NanoDrop™ spectrophotometer and was converted to the number of molecules by using Avogadro’s constant.

A 10-fold serial dilution was prepared for plasmid concentrations, ranging from 2.4 × 10^2^ to 2.4 × 10^9^ copies/μL. Four replicate serial dilutions were then used in real-time PCR to generate a standard curve against the threshold cycle. The amplification efficiency of the standard curve was calculated by using the formula 10^(−1/slope of the standard curve)^.

### 4.5. RT-qPCR for the Absolute Quantification of Viral RNA

Real-time PCR was conducted using a SensiFAST SYBR No-ROX kit (Bioline, London, UK) according to the instructions for Rotor-Gene^TM^ 6000 (Qiagen, Hilden, Germany). The cycling conditions were 95 °C for 2 min, followed by 40 cycles of 95 °C for 5 s, 61 °C for 10 s, and 72 °C for 10 s. The cDNA samples were added to a final concentration of 20 ng/µL. The serial dilutions of plasmid DNA were added for standard curve generation, and the primers CaCV_IR_qPF and CaCV_IR_qPR (Table 2) were designed from the cloned CaCV IR target sequence. A selected cDNA sample was included in each run as a control for normalizing the Ct values using Rotor-Gene 6000 software v1.7. A linear regression curve plot and a standard curve for each run were also generated using Rotor-Gene software.

### 4.6. RT-qPCR for Gene Expression Analysis

Defense-associated capsicum genes were selected on the basis of their known involvement in plant defense responses. Primers were designed from the transcript sequences obtained from our previous study (Widana Gamage et al., 2016) and are listed in Table 2. *Rg2* (transcript ID 10552), *SGT1* (ID 17234), and *PIF4* (ID 33954) were selected based on R gene-related defense functions. *DCL2* (ID 50375), *DCL4* (ID 31955), *AGO1a*, (ID 28576), *AGO1b* (ID 16835), *AGO2* (ID 11920), *RdRp1* (ID 34640), and *RdRp6* (ID 25145) were selected based on RNAi-related defense functions. The transcript levels of those genes were quantified relative to the *Actin* gene (ID 48353). Total RNA isolation, DNase treatment, cDNA synthesis, and real-time PCR were conducted using the methods described in Section 4.3 above. To identify a potential contamination affecting the results of the real-time PCR, no amplification or more than five cycles difference and melting curve difference in no-template samples and no-RT samples compared to unknown samples were considered as indicators. The relative gene expression was calculated by the 2^−ΔCt^ method [71].

### 4.7. Northern Blot Hybridization for Detecting Virus-Derived siRNAs

Low molecular weight (LMW) RNA was isolated following the method described in [72]. Briefly, polyethylene glycol was added to a final concentration of 7.5% to precipitate high molecular weight RNA. Subsequently, the supernatant was transferred and mixed with a 2/3 volume of absolute ethanol and a 1/10 volume of 3M sodium acetate to precipitate LMW RNA at −20 °C overnight. LMW RNA was collected by centrifugation at 15,300× *g* at 4 °C for 10 min and air-dried. LMW RNA (2 μg) was electrophoresed through a 18% (wt/vol) polyacrylamide gel under denaturing conditions (8 M urea). MicroRNA labelled with digoxigenin (DIG) at the 3′ end (New England BioLabs, Ipswich, MA, USA) was used as a marker. The gel-separated RNAs were transferred onto a Hybond-N+ nylon membrane (Roche, Mannheim, Germany) using a Bio-Rad trans-blot SD semi-dry transfer unit. U6 (5′-TCATCCTTGCGCAGGGGCCA) was DIG-labelled using the DIG Oligonucleotide 3′-End Labelling Kit. The probe for detecting vsiRNA was amplified using a PCR DIG probe synthesis kit (Roche, Mannheim, Germany) with primers (F:5′-GACAACAGCAATGAAAGAACAACT and R:5′-AATCCTCCCCGAAACTTCCAC) designed to amplify a fragment of the CaCV S RNA segment. The relative accumulation levels of the vsiRNA were estimated by densitometry of northern blot images using ImageJ software (Version 1.52q) (https://imagej.nih.gov/ij/download.html accessed on 27 January 2022). The densitometry value of HT/CaCV at 18 dpi was used for normalization and set as 1 in each blot. Subsequently, the normalized U6 values were set as standards to normalize the normalized values of the vsiRNAs.

### 4.8. Statistics

Data were analyzed with the unpaired Student’s *t*-test using GraphPadPrism software (Version 8.4.3, San Diego, CA 92108, USA). The nonparametric or parametric test and their corresponding correction methods were selected based on the test result of normal distribution and equal variance in each data set. The differences were considered to be significant if the two-tailed *p* value was <0.05. A correlation between the abundance of vsiRNAs and gene expression over time was calculated using the repeated measures correlation (rmcorr) method [73]. The rmcorr package in R (Version 3.6.3) was used for computing and plotting the within-individual association that was assessed at three time points for four biological replicates.

## Figures and Tables

**Table 1:** Symptom scores of CaCV-infected *C. annuum* cv. Yolo Wonder plants.

Scores	Visible Symptoms
0	No visible symptoms; inoculated plants show the same growth and development as mock-inoculated plants.
1	Very slight yellowing in up to 25% of the inoculated leaf area; the same development as mock-inoculated plants.
2	Some chlorotic spots in up to 50% of the inoculated leaf area; the same development as mock-inoculated plants.
3	Chlorotic spots and/or interveinal chlorosis in more than 50% of the inoculated leaf area; chlorotic spots are starting to show on systemic leaves.
4	Leaf curling and leaf rugosity on systemic leaves.
5	Strong curling and rugosity; mild stunting.
6	Very strong leaf curling and rugosity, with chlorotic spots in all systemic leaves; severe plant stunting.

**Table 2:** Primers for the absolute quantification and RT-qPCR gene expression analysis of selected capsicum defense-related genes.

Primer Name	Primer Sequence (5′ to 3′)
Ca_Rg2_qPF	AGGTAAAAGAATTATATCTCAA
Ca_Rg2_qPR	TGCAGAGGGTTTGTAGGCTT
Ca_SGT1_qPF	GATCCTCAATCAACTGTCAACCTG
Ca_SGT1_qPR	CCCTTGGCAAATATAGTCACAACC
Ca_PIF4_qPF	AAAGGAAAAGCAGAGATGGTGAAGA
Ca_PIF4_qPR	CCTTTCAGAGAGGTTATGCACTTC
Ca_DCL2_qPF	TGGTTATGGCCTCGAACTTGA
Ca_DCL2_qPR	TCCCCAAGTGTCTCAAGTGAT
Ca_DCL4_qPF	CCAATCTGTACATGGTAGCAGTC
Ca_DCL4_qPR	TGTGCTTGTTACAGGTTACAGG
Ca_RdRp6_qPF	CCTCTACTTTGTGACTTGGGATGA
Ca_RdRp6_qPR	TGTGCGTTGCAGATTTCTCCT
Ca_RdRp1_qPF	ATGCAGAGGCCATTGGTGTTGCTG
Ca_RdRp1_qPR	CCAAGCTGAAGCCTTTGGTAACAT
Ca_AGO1a_qPF	AAACTATTTGCCAATGGAGGTCTG
Ca_AGO1a_qPR	ACAGTCTGAAGAATATCACCCTCTC
Ca_AGO1b_qPF	AACAAGAGAGTTGACTTTTCCTGT
Ca_AGO1b_qPR	CAAGTAATTAGGTCGCTGCTGATT
Ca_AGO2_qPF	TCGTCTGATCCTGTTCAAGTTGAT
Ca_AGO2_qPR	CTGACTTAACAGCAATTTTTCCAGT
Ca_actin_qPF	CCCTAAGGCCAACAGAGAGAA
Ca_actin_qPR	CTCACACCATCACCAGAGTCC
CaCV IRF	CACTCATTGTTTGCATGCTGGAA
CaCV IRR	ACACTAAAGCTTTGAGAGAAGTTAG
CaCV IR_qPF	GCTTGTACATTTAGTTTATCAGGGTTAG
CaCV IR_qPR	CCAATTTGTTGAATGAGCTAACTTTGG

## Data Availability

Not applicable.

## Supplementary Materials

The following are available online at https://www.mdpi.com/article/10.3390/pathogens11020200/s1, Figure S1: Quantitative PCR standard curves of ten-fold serial dilutions of plasmid DNA containing capsicum chlorosis virus (CaCV) S segment intergenic region, Figure S2: Scatter plots for the repeated measures correlations between the expression of RNA interference-associated genes and the abundance of virus-derived short-interfering RNAs at high temperature over time, Figure S3: Effect of temperature on capsicum chlorosis virus (CaCV) infection in *Nicotiana benthamiana* laboratory line (LAB) and *N. benthamiana* Western Australia line (WA).
