# Supplementary material for: Effects of Elevated Temperature on the Susceptibility of Capsicum Plants to Capsicum Chlorosis Virus Infection
Source: Pathogens. 2022 Feb 2;11(2):200. doi: 10.3390/pathogens11020200 (PMC8879237; doi:10.3390/pathogens11020200)
Supplement: Supplementary file 1 [file pathogens-11-00200-s001.zip › supplementary/Legends for supplementary figures_vi3-RD.docx]

**Figure S1** Quantitative PCR standard curves of ten-fold serial dilutions of plasmid DNA containing capsicum chlorosis virus (CaCV) S segment intergenic region. Accumulation of CaCV RNA was measured by absolute quantification in inoculated leaves at 5 dpi and in systemic leaves at 10 and 18 dpi. Ct values were plotted against the corresponding log values of virus concentration (copy number/ul). R^2^ = 0.99583; the amplification efficiency of real-time PCR was 98%. The blue dots in the upper panel represent the ten-fold serial dilutions for standard curve construction and the red dots in both upper and lower panels represent the test samples.

**Figure S2**Scatter plots for repeated measures correlations between expression of RNA interference - associated genes: (A) *DCL2*, (B) *DCL4*, (C) *RdRp1*, (D) *RdRp6*, (E) *AGO1a*, (F) *AGO1b*, and (G) *AGO2* and abundance of virus-derived short-interfering RNAs (vsiRNAs) at high temperature (HT) over time. Different colors represent different individuals of 4 biological replicates. R values represents a strong positive correlation when close to 1, and a negative correlation when close to -1. Significant correlation is indicated if P value is < 0.05.

**Figure S3** Effect of temperature on capsicum chlorosis virus (CaCV) infection in *Nicotiana benthamiana* laboratory isolate (LAB) and *N. benthamiana* Western Australia isolate (WA). Representative symptoms of CaCV-infected WA and LAB plants grown at ambient temperature (AT) of 25°C (pictures on the right) in contrast to those plants mock-treated with buffer (pictures on the left) at (A) 10 days post inoculation (dpi), and (C) at 20 dpi. Representative symptoms of CaCV-infected WA and LAB plants grown at high temperature (HT) of 35°C (right) in contrast to those plants mock-treated with buffer (left) at 10 dpi (B), and at 20 dpi (D).
