# Supplementary figures and images for: Effects of Elevated Temperature on the Susceptibility of Capsicum Plants to Capsicum Chlorosis Virus Infection
Source: Pathogens. 2022 Feb 2;11(2):200. doi: 10.3390/pathogens11020200 (PMC8879237; doi:10.3390/pathogens11020200)

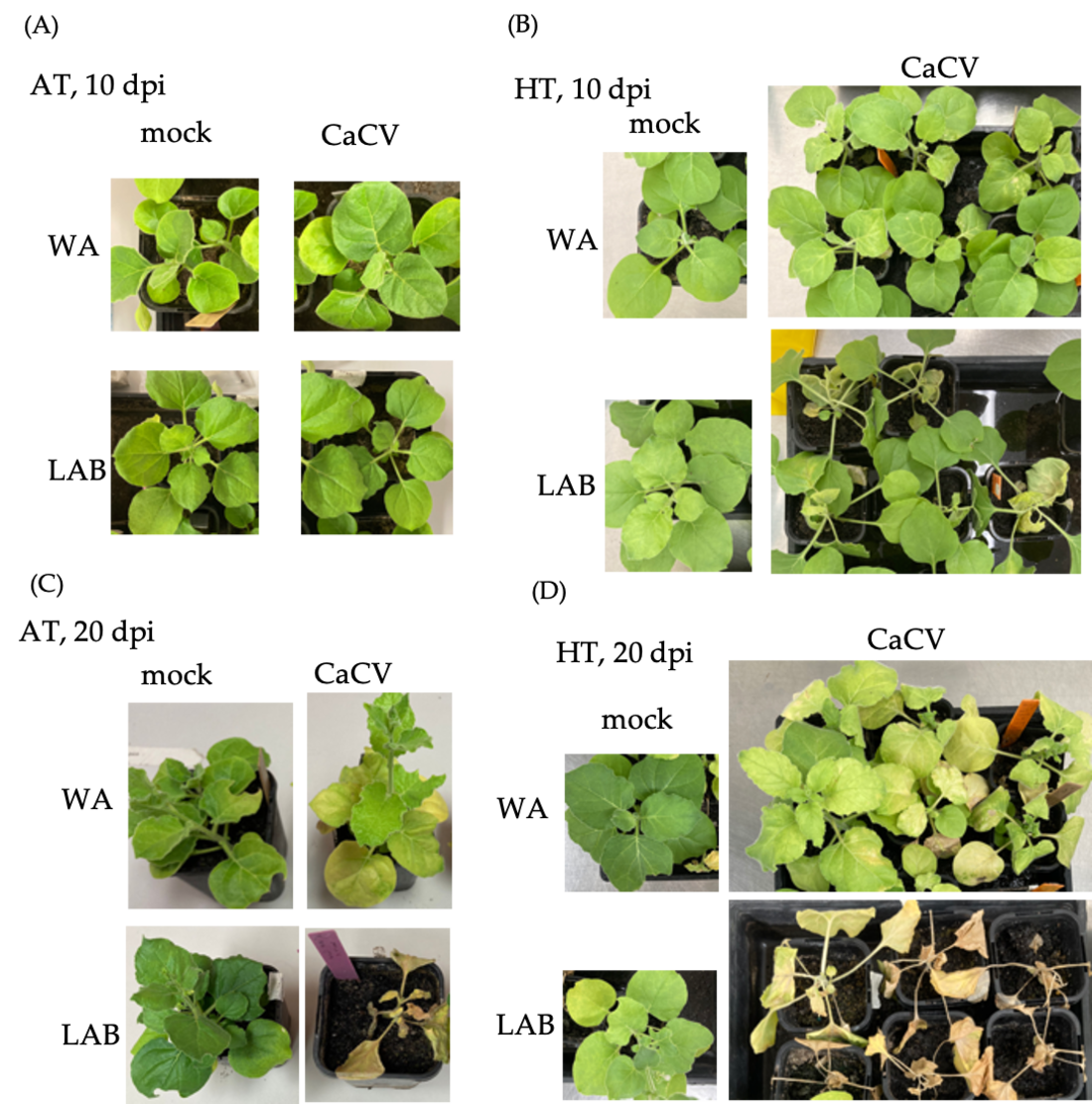

Supplement: Supplementary file 1 [file pathogens-11-00200-s001.zip › supplementary/Fig.S3.png]

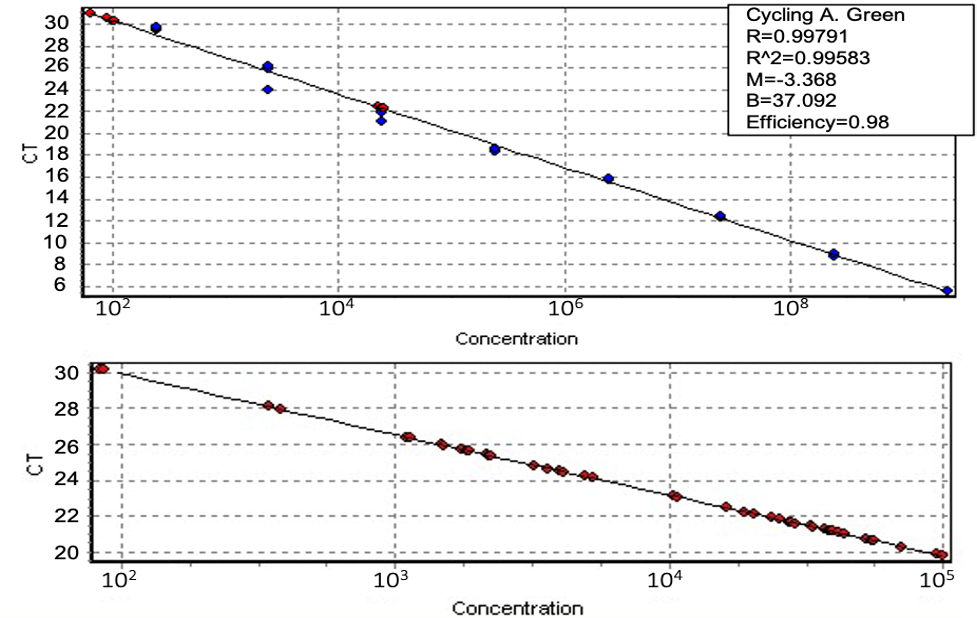

Supplement: Supplementary file 1 [file pathogens-11-00200-s001.zip › supplementary/Figure.S1.png]

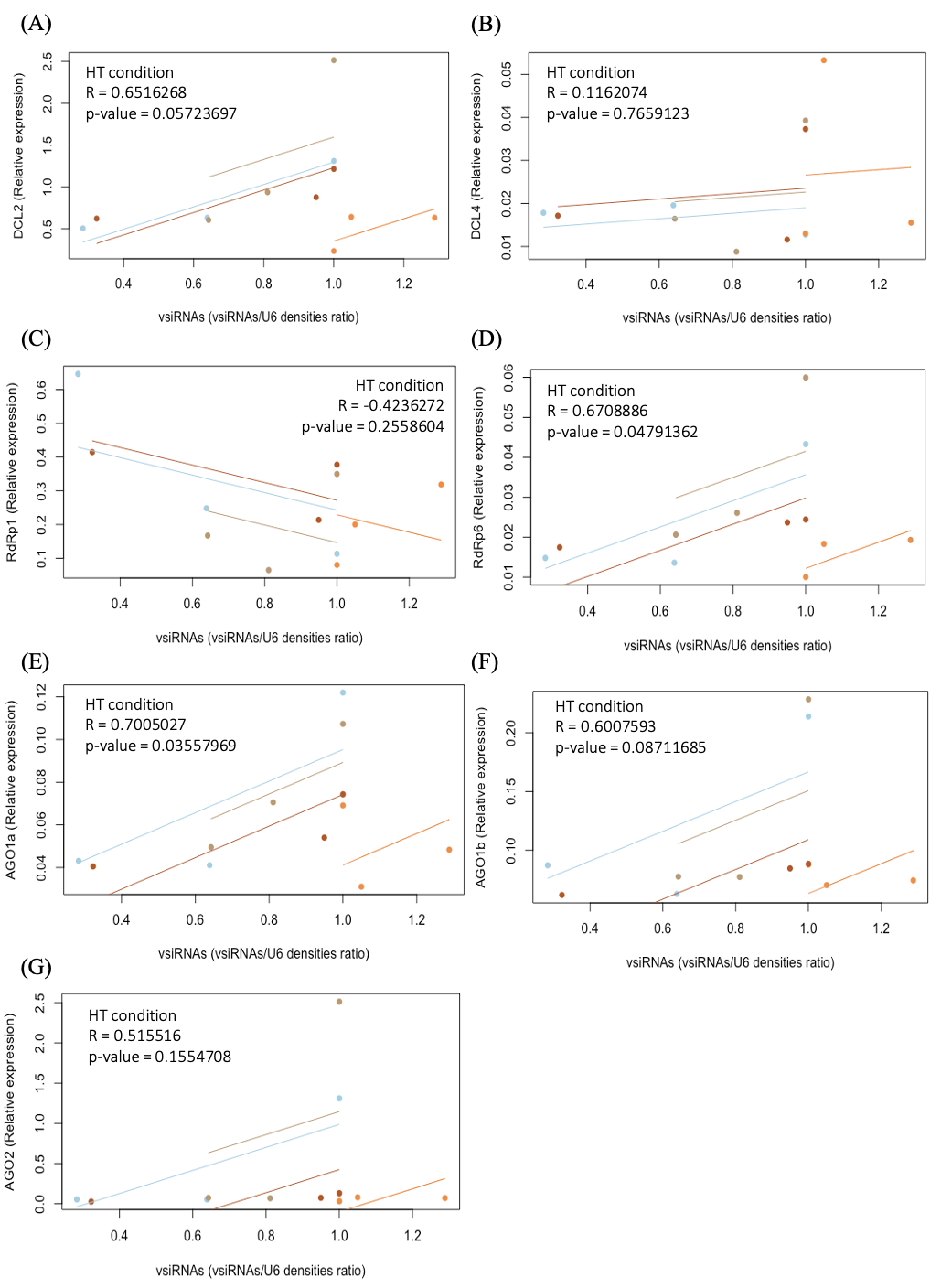

Supplement: Supplementary file 1 [file pathogens-11-00200-s001.zip › supplementary/Figure.S2.png]
